# Climate Variability, Social and Environmental Factors, and Ross River Virus Transmission: Research Development and Future Research Needs

**DOI:** 10.1289/ehp.11680

**Published:** 2008-07-24

**Authors:** Shilu Tong, Pat Dale, Neville Nicholls, John S. Mackenzie, Rodney Wolff, Anthony J. McMichael

**Affiliations:** 1 School of Public Health and Institute of Health and Biomedical Innovation, Queensland University of Technology, Kelvin Grove, Australia; 2 Griffith School of Environment, Griffith University, Nathan, Australia; 3 School of Geography and Environmental Science, Monash University, Melbourne, Australia; 4 Australian Biosecurity CRC, Curtin University of Technology, Perth, Australia; 5 School of Mathematical Sciences, Queensland University of Technology, Gardens Point, Australia; 6 National Centre for Epidemiology and Population Health, Australian National University, Canberra, Australia

**Keywords:** climate variability, early warning system, Ross River virus transmission, social and environmental factors

## Abstract

**Background:**

Arbovirus diseases have emerged as a global public health concern. However, the impact of climatic, social, and environmental variability on the transmission of arbovirus diseases remains to be determined.

**Objective:**

Our goal for this study was to provide an overview of research development and future research directions about the interrelationship between climate variability, social and environmental factors, and the transmission of Ross River virus (RRV), the most common and widespread arbovirus disease in Australia.

**Methods:**

We conducted a systematic literature search on climatic, social, and environmental factors and RRV disease. Potentially relevant studies were identified from a series of electronic searches.

**Results:**

The body of evidence revealed that the transmission cycles of RRV disease appear to be sensitive to climate and tidal variability. Rainfall, temperature, and high tides were among major determinants of the transmission of RRV disease at the macro level. However, the nature and magnitude of the interrelationship between climate variability, mosquito density, and the transmission of RRV disease varied with geographic area and socioenvironmental condition. Projected anthropogenic global climatic change may result in an increase in RRV infections, and the key determinants of RRV transmission we have identified here may be useful in the development of an early warning system.

**Conclusions:**

The analysis indicates that there is a complex relationship between climate variability, social and environmental factors, and RRV transmission. Different strategies may be needed for the control and prevention of RRV disease at different levels. These research findings could be used as an additional tool to support decision making in disease control/surveillance and risk management.

The global climate is changing at an unprecedented rate, with rising surface temperatures, melting glaciers, rising sea levels, and changes in climate variability and extreme events. Questions about its possible public health consequences command increasing attention. It has become evident that global climate change is altering—indeed, will continue to alter—the pattern of both average climatic conditions and variability, although the projected trends within specific geographic regions remain uncertain (Intergovernmental Panel on Climate Change 2007; Royer et al. 2007). There are unresolved questions about *a*) how climate change may affect the transmission of vector-borne diseases, and *b*) to what extent the empirical evidence of short-term climate variation and disease transmission can be applied to the estimation of future impacts of climate change.

Arboviral infections are a global health problem accounting for significant morbidity and mortality in human populations (Ansari and Shope 1994; Gubler 2002). Ross River virus (RRV) infection is the most common and widespread arboviral disease in Australia and some Pacific island nations, with thousands of clinical cases occurring each year [Australian Department of Health and Ageing (ADHA) 2008; Mackenzie et al. 1998]. Over the last 15 years (i.e., 1993–2007), 64,562 laboratory-confirmed RRV cases (4,304/year) have been reported in Australia alone (ADHA 2008). Of these, more than half were reported in Queensland, a subtropical and tropical state. In recent decades, RRV outbreaks have increased, notably in urban estates and tourist destinations (Harley et al. 2001; Kelly-Hope et al. 2004a; Tong et al. 2001). For example, in Australia, there were 8,387 notifications of mosquito-borne diseases (MBDs) during the period 1 July 2005 to 30 June 2006, which was the second highest on record since 1995–1996 (Liu et al. 2006). RRV infections accounted for about 70% of MBD notifications with an onset in 2005–2006. The major behavioral, environmental, and ecologic risk factors for RRV disease transmission include outdoor activities (e.g., camping), climate variability (e.g., increasing rainfall and temperature), people moving from nonendemic into endemic areas, and the effectiveness of vector control programs. The reasons for the increased RRV activity may include urban developments in or near wetland and salt marsh habitats, increased travel, and socioecologic changes (Muhar et al. 2000; Russell 2002; Tong et al. 2004).

Because RRV infection is the most common and widespread mosquito-borne disease in Australia, there has been an increasing research interest in the assessment of major determinants of RRV transmission (Hu et al. 2004, 2006a, 2006b; Jacups et al. 2008, Kelly-Hope et al. 2004a; Tong et al. 2002, 2005; Woodruff et al. 2006). To identify current knowledge gaps and research needs, we critically reviewed the impact of climatic, social, and environmental variability on the transmission of RRV disease. Here we provide an overview of research development and future research directions in this emerging field.

## Characteristics and Ecology of Ross River Virus Disease

RRV causes a nonfatal but prolonged and debilitating disease known as epidemic polyarthritis or RRV disease. The disease syndrome is characterized by headache, fever, rash, lethargy, and muscle and joint pain. In most cases, the symptoms persist for 3–6 months and can be severe (Harley et al. 2001; Mackenzie et al. 1998), but like the other arthritogenic alphaviruses, occasional cases may persist for longer periods (Condon and Rouse 1995; Smith et al. 2008).

The annual costs of symptom management and productivity losses are estimated at $3–6 million in Australia, although this sum does not account for public health surveillance, control and response activities, or full diagnostic and medical costs (Harley et al. 2001; Hawkes et al. 1985). Recently, more detailed evaluation has shown economic costs at an individual level of > $1,000 on average, which can render a significant economic burden to infected residents, particularly poor people (Ratnayake 2005). Outbreaks of RRV impact considerably on tourism and industry, as well as on communities (Harley et al. 2001; Hawkes et al. 1985; Mackenzie et al. 1998). Additionally, there is substantial expenditure for vector control programs each year (e.g., > $10 million in Queensland in 2004 alone) (Tomerini 2007). Further, there is an increasing concern about the risk of introducing RRV to neighboring countries such as New Zealand (Kelly-Hope et al. 2002) and the global emergence/resurgence of arbovirus diseases (Gubler 2002). Therefore, it is important to improve the public health strategy to control and prevent this widespread disease in the Oceania region (Guillaumot 2005; McMichael et al. 2003).

The ecology of RRV is complex. The virus and its reservoir host species, the vector, the human population, and environmental conditions play key roles in the RRV transmission cycles. RRV is an alphavirus that was named after it was first isolated from *Ocherotatus vigilax* mosquitoes collected around Ross River near Townsville in northern Queensland in 1959 (Doherty et al. 1963). The natural vertebrate hosts for this disease include marsupial animals (e.g., kangaroos, wallabies) and possibly other animals (e.g., dogs, cats, horses, possums). The incubation period may be as long as 21 days or as short as 3 days (usually 7–9 days) (Harley et al. 2002). More than 30 mosquito species have been implicated as vectors of RRV (Mackenzie et al. 1994; Russell 1994). *Ocherotatus vigilax* and *Ocherotatus camptorhynchus*, which breed in intertidal wetlands, are important in coastal regions, as are floodwater *Aedes* species in many inland areas. *Culex annulirostris*, which breeds in vegetated semipermanent and permanent fresh water, is common in areas of the tropics and temperate regions that are subject to flooding or irrigation during summer. Species such as *Ocherotatus notoscriptus* may be important in semirural and urban areas (Harley et al. 2002; Mackenzie et al. 1994; Russell 1994).

The virus is dependent on the continuing recruitment of nonimmune hosts in the reservoir population. The distribution and abundance of the reservoir population will affect the availability of viremic individuals to mosquitoes, and a nonimmune reservoir population leads to increased virus activity (Weinstein 1997). A number of mosquito-related factors (e.g., mosquito species, mosquito abundance) also influence the level of RRV activity. The human population is susceptible to RRV infection if individuals are nonimmune and are exposed to the virus at the reservoir/mosquito/human interface. Such exposure is enhanced by human intrusions into natural ecosystems by the expansion of agriculture, forestry, tourism, and building of new suburbs in or near wetlands (Russell 2002; Weinstein 1997).

**Figure 1:**
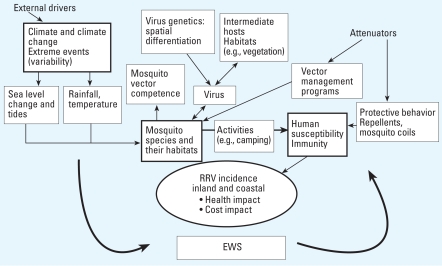
Interrelations between climate variability, social and environmental factors, and RRV transmission.

Weather conditions directly affect the breeding, survival, and abundance of mosquitoes and the extrinsic incubation period of the disease. In seasons with high temperatures and rainfall, the vegetation upon which intermediate hosts such as kangaroos, possums, and horses depend will flourish, and more nonimmune reservoir hosts will be added to the temporally and spatially expanding population (Harley et al. 2001; Hu et al. 2004; Kelly-Hope et al. 2004a; Russell 2002). RRV disease has strong spatial and temporal patterns, because mosquito density and longevity depend on a number of environmental and social factors (e.g., temperature, precipitation, mosquito-breeding habitats, vector control programs) (Gatton et al. 2005; Hu et al. 2005; Tong et al. 2001). RRV is considered to be one of the few infectious diseases that can be predicted by climate-based early warning systems (EWS) [World Health Organization (WHO) 2004], although predictions may vary because of local conditions (Mackenzie et al. 2000). Climate variability strongly affects the replication of the virus; the breeding, abundance, and survival of the mosquito species; the breeding patterns of major hosts; and people’s behavior (Harley et al. 2001; Tong et al. 2004). These variables can be modeled to predict the onset and severity of disease epidemics at different temporal and spatial scales (Hightower et al. 1998; Moore and Carpenter 1999). However, in developing EWS for MBDs and other infectious diseases, it is important to also include nonclimatic influences in the models and to make the models relevant to particular response decisions and to the needs of policy makers (WHO 2004). Below we discuss exemplary research on the complex interactions relevant to developing an EWS (Figure 1).

## Climate Variability, Social and Environmental Factors, and RRV Transmission

### Identifying relevant studies

To identify all relevant studies (in addition to our own research), we conducted a systematic literature search on climatic, social, and environmental factors and RRV disease. Potentially relevant studies were identified from a series of electronic searches. Databases searched were MEDLINE (via EBSCOhost; EBSCO Publishing, Ipswich, MA), Current Contents Connect (via ISI Web of Knowledge; Thomson Reuters, London, UK), and ScienceDirect (Elsevier B.V., Amsterdam, the Netherlands). Literature searches were conducted in November 2007, with no date or language restriction. The databases were searched with the following key words or MeSH terms: “Ross River virus” AND “climate” OR “social” OR “environmental” OR “climatic” OR “rainfall” OR “temperature.” In addition, in June 2008 we performed a further complementary search of articles published since 2007. Titles and abstracts for all the publications identified by the literature search were first assessed for suitability for inclusion. Full copies of all suitable studies were then obtained and critically reviewed.

### Key predictors of RRV transmission: integration of our own research with literature

The primary objectives of our own research were to assess the impact of climate variability and social and environmental factors on the transmission of RRV disease, and their role in developing EWS for the control and prevention of this most common and widespread MBD in Australia. We also identified several other studies on this topic through the literature search (Table 1).

**Figure 2:**
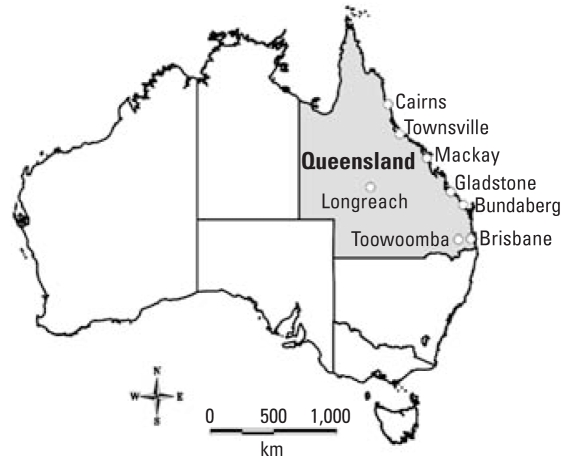
Location of Queensland, Australia (including eight major cities).

We collected a large amount of data from relevant government agencies on climate variables, social and environmental factors, and notified cases of RRV disease for Queensland, where most cases are usually reported (Figure 2). A range of geographic information system (GIS) techniques and ecologic time-series models were performed to assess the impact of climate variability on RRV transmission in Queensland (Hu et al. 2005, 2006a, 2006b, 2007; Tong et al. 1998, 2001, 2002, 2004, 2005; Tong and Hu 2002).

**Figure 3:**
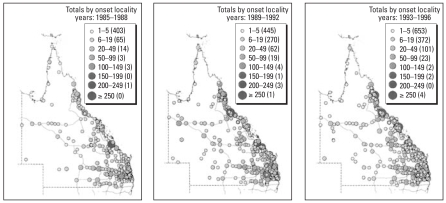
Geographic distribution of notified RRV cases in Queensland, Australia, 1985–1996. Numbers in parentheses indicate the number of localities. Adapted from Tong et al. (2001).

In a previous study, we used GIS techniques to assess the distribution of RRV in Queensland (Tong et al. 2001). For that study, we obtained the computerized data set on the notified RRV cases in Queensland for the period of 1985–1996 and key sociodemographic information from Queensland Health (Brisbane, Queensland, Australia). The reported place of onset for each case was used to characterize the geographic distribution of the RRV infection within Queensland. We used MapInfo Professional (Ersis Australia, Sydney, Australia) to display the spatial and temporal distributions of RRV cases. The digital base map data sets used for constructing the GIS were obtained primarily from the Queensland Department of Natural Resources (Brisbane, Queensland, Australia). The onset places in the data set were geocoded to the digital base maps of localities using MapInfo and Microsoft Access (Microsoft Corporation, Redmond, WA, USA) software. The location for each notified case of the RRV infection was then obtained by overlaying the database of the onset places of notified RRV infections with the digital base maps. In that study (Tong et al. 2001), we found RRV notifications from 489 localities between 1985 and 1988. By the period 1989–1992, this had increased by 65% to 805 localities and, by 1993–1996, it had increased to 1,157 localities, an increase of 137% from the 1985–1988 period (Figure 3). The geographic distribution of the notified RRV cases has apparently expanded in Queensland over recent years, as shown in Figure 3, and it is unlikely to be entirely explained by better notification and laboratory reporting, because the geographic distribution of RRV varied substantially in Queensland. That study (Tong et al. 2001) was the first to use GIS techniques to display the variation of the detailed spatio-temporal distribution of RRV disease. The study suggested that the geographic expansion of RRV may be associated with many factors, including population growth, urban sprawl, increased travel, and socioecologic change.

In another study (Tong et al. 2002), we performed an ecologic time-series analysis to examine the association between climate variability and the transmission of RRV disease between 1985 and 1996 in Queensland. Information on the notified RRV cases was obtained from the Queensland Health, and climate and population data were supplied by the Australian Bureau of Meteorology and the Australian Bureau of Statistics, respectively. The autoregressive integrated moving average model was used to examine the relation between climate variability, tides, and the monthly incidence of notified RRV infections. As maximum and minimum temperatures were highly correlated with each other (*r*_s_ = 0.75), two separate models were developed. For the eight major cities in Queensland, the climate–RRV correlation coefficients were in the range of 0.12–0.52 for maximum and minimum temperatures, –0.10 to 0.46 for rainfall, and 0.11–0.52 for relative humidity and high tide (Tong et al. 2002). For the whole state, rainfall [partial regression coefficient, 0.017 (95% confidence interval, 0.009–0.025) in Model I and 0.018 (0.010–0.026) in Model II], and high tidal level [0.030 (0.006–0.054) in Model I and 0.029 (0.005–0.053) in Model II] seemed to have played significant parts in the transmission of RRV in Queensland. Maximum temperature was also marginally significantly associated with the incidence of RRV infection. The results of our research (Tong et al. 2002) showed that although many factors can affect the RRV transmission cycles, RRV disease is generally sensitive to climate variability. At a macro level, rainfall, temperature, and tidal levels appeared to be important environmental determinants in the transmission cycles of RRV disease. The findings are generally consistent with other studies (Hu et al. 2005, 2006a, 2007; Kelly-Hope et al. 2004a; Mackenzie et al. 2000; Russell 2002; Tong et al. 2002, 2004, 2005; Tong and Hu 2002). This information may be useful for designing and implementing RRV interventions at the state and national levels.

However, the magnitude and nature of the association between climate variables and social and environmental factors and RRV transmission seemed to vary with geographic area (Kelly-Hope et al. 2004b; Tong and Hu 2002). For example, we assessed the difference in the potential predictors of the RRV incidence in coastline and inland regions of Queensland using time-series regression models (Tong and Hu 2002). The function of cross-correlations was used to compute a series of correlations between climate variables (rainfall, maximum temperature, minimum temperature, relative humidity, and high tide) and the monthly incidence of RRV disease over a range of time lags. In that study (Tong and Hu 2002), time-series regression models were performed to adjust for the autocorrelations of the monthly incidences of RRV disease and the confounding effects of seasonality, the case notification time, and population sizes. The results showed that the incidence of RRV disease was significantly associated with rainfall, maximum temperature, minimum temperature, relative humidity, and high tide in the coastline region, and with rainfall and relative humidity in the inland region. There was a significant interaction between climate variables and locality in RRV transmission. Different responses of RRV to climate variability were observed between coastal and inland cities in Queensland (Tong and Hu 2002). Overall, rainfall appeared to be the single most important determinant of RRV transmission. However, maximum temperature appeared to exhibit greater impacts on the RRV transmission in coastal cities than in inland cities. Minimum temperature and relative humidity seemed to affect the RRV transmission inland more than at the coast.

Kelly-Hope et al. (2004b) also compared seasonal and monthly rainfall and temperature trends in outbreak and nonoutbreak years at four epidemic-prone locations in Australia. Their analyses showed that rainfall in outbreak years tended to be above average and higher than rainfall in nonoutbreak years. Overall temperatures were warmer during outbreak years. However, there were a number of distinct deviations in temperature, which seemed to play a role in either promoting or inhibiting outbreaks (Kelly-Hope et al. 2004b). These results showed that climatic differences occur between outbreak and nonoutbreak years; however, seasonal and monthly trends differed across geoclimatic regions of the country.

Now, considerable evidence has accrued to show that precipitation is an important factor in the transmission of RRV in many parts of Australia. All mosquitoes have aquatic larval and pupae stages and therefore require water for breeding (Hawkes et al. 1985; Muhar et al. 2000). It is precipitation that determines the presence or absence of breeding sites. Rainfall events and subsequent floods can lead to outbreaks of arboviral disease, largely by enabling breeding of vector mosquitoes (Hawkes et al. 1985). In general, epidemic activity of arbovirus is more often observed in temperate areas with heavy rainfall, flooding, or high tides, whereas in tropical Australia transmission occurs throughout the year (Russell 2002).

**Figure 4:**
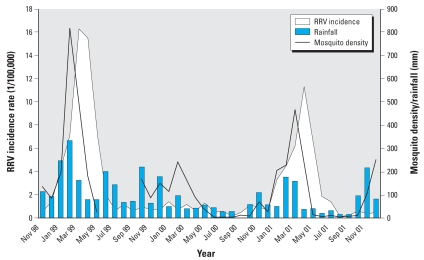
Mosquito density, rainfall, and RRV in Brisbane for November 1998–December 2001. Adapted from Hu et al. (2006a).

A number of studies have attempted to develop predictive models for the transmission of RRV disease using routinely collected data on climate and tidal variables and mosquito and disease surveillance (Hu et al. 2006a; Tong et al. 2005; Woodruff et al. 2006). Our recent research at the city level reveals that in some areas (e.g., the Brisbane metropolitan area), rainfall and sea tides directly influence the density of mosquitoes such as *Culex annulirostris* and *Aedes vigilax*, respectively, which then affects the transmission patterns of RRV in inland and coastal suburbs (Hu et al. 2006a; Tong et al. 2005). For Brisbane City, we obtained monthly data on the counts of RRV cases, monthly total rainfall, human population size, and mosquito density (i.e., average number of mosquitoes trapped in all mosquito-monitoring stations per month) between 1 November 1998 and 31 December 2001 from the Queensland Department of Health, Australian Bureau of Meteorology, Australia Bureau of Statistics, and Brisbane City Council, respectively. Both polynomial distributed lag time-series regression and seasonal auto-regressive integrated moving average (SARIMA) models were used to examine associations of RRV transmission with rainfall and mosquito density after adjustment for seasonality and auto-correlation. The results show that 85% and 95% of the variance in the RRV transmission was accounted for by rainfall and mosquito density, respectively (Hu et al. 2006a). Both rainfall and mosquito density were strong predictors of the RRV transmission in simple models. However, multivariate polynomial distributed lag models show that only mosquito density at lags of 0 and 1 month was significantly associated with the transmission of RRV disease. The SARIMA models show similar results (Figure 4) (Hu et al. 2006a).

Woodruff et al. (2006) focused on a temperate region of western Australia between July 1991 and June 1999. Both early and later warning logistic regression models were developed to test the sensitivity of data on environment (tide height, rainfall, sea surface temperature) and mosquito counts for predicting epidemics of disease. Environment data alone were moderately sensitive (64%) for predicting epidemics during the early warning period. Addition of mosquito surveillance data increased the sensitivity of the early warning model to 90%. The later warning model had a sensitivity of 85%. Woodruff et al. (2006) concluded that the environment data they used are relatively cheap to collect and useful for the prediction of RRV disease epidemics. Mosquito surveillance data provide a more expensive early warning but add substantial predictive value.

Recently, Jacups et al. (2008) described the epidemiology of RRV infection in the endemic Darwin region of tropical northern Australia and developed a predictive model for RRV infections. Laboratory-confirmed cases of RRV infection between 1 January 1991 and 30 June 2006 were analyzed, together with climate, tidal, and mosquito data collected weekly over the study period from 11 trap sites around Darwin, using both correlations and Poisson regression models. Correlations revealed strong associations between monthly RRV infections and climatic variables and also each of the four implicated mosquito species populations. The best global model included rainfall, minimum temperature, and three mosquito species, which explained 63.5% deviance and predicted disease accurately. The results also indicate that predicted anthropogenic global climatic changes may result in an increase in RRV infections (Jacups et al. 2008).

These research findings provide further evidence that mosquito density, largely influenced by rainfall and high tide, is an important risk factor for RRV transmission. Increased mosquito density increases the likelihood that a person will be bitten and, hence, this affects the risk of contracting a mosquito-borne disease. Improved understanding of the relationship between environmental variability, mosquito density, and RRV transmission might assist disease control managers in planning and implementing public health interventions. Results of studies such as this might facilitate the development of EWS for reducing the incidence of this widespread disease and other MBDs in Australia and other Pacific island nations.

## Other Risk Factors

### Individual and behavioral risk factors

Harley et al. (2005) examined individual risk and protective factors for RRV disease in a high-incidence area. They performed a prospective matched case–control study with new community cases of RRV disease in the local government areas of Cairns, Mareeba, Douglas, and Atherton, in tropical Queensland, from 1 January to 31 May 1998. Protective measures against mosquitoes reduced the risk for disease. Mosquito coils, repellents, and citronella candles each decreased risk by at least 2-fold, with a dose response for the number of protective measures used. Light-colored clothing decreased risk 3-fold, and camping increased the risk 8-fold (Harley et al. 2005). These risks were substantial and statistically significant, and provide a basis for educational programs on individual protection against RRV disease in Australia.

### Genetic divergence

Sammels et al. (1995) conducted a molecular epidemiologic study to examine the evolution of RRV in Australia and the Pacific Islands. Nucleotide sequences of the *E2* and *E3* genes of five RRV strains revealed remarkable conservation between 1959 and 1989, with a maximum divergence of only 3.3%. Sequence data from a 505-base pair fragment of the *E2* gene from 51 additional strains showed that RRV has diverged genetically into three separate groups, although at least 95% sequence homology was still maintained between all 56 strains. Each genetic type predominates in a particular geographic region of Australia and can be broadly defined as occurring in the western, northeastern, and southeastern regions of Australia. However, some RRV strains did not follow this pattern of geographic distribution, indicating movement of virus by the travel of viremic humans or livestock across the continent. The Pacific Islands isolates all belong to the southeastern genotype. The findings of Sammels et al. (1995) suggest genetic divergence and independent evolution of RRV within geographically isolated enzootic foci; however, selective pressures maintain high nucleotide conservation in nature.

## Vector Control Programs

As outbreaks of RRV impact considerably on tourism and industry, as well as on communities (Harley et al. 2001; Russell 2002), there is substantial expenditure for vector control programs each year (e.g., > $10 million in Queensland in 2004 alone) (Tomerini 2007). Tomerini also showed that the costs of mosquito control may be offset by reduced incidence (and cost) of RRV disease. There is a need to examine how to undertake vector control programs more effectively and efficiently. For example, more resources should be mobilized into high-risk areas during high-risk periods but fewer resources are needed in low-risk areas or during nonepidemic periods. At present, a similar amount of funding is provided to implement vector control activity in most areas each year regardless of epidemic or nonepidemic circumstances.

## Development of EWS

EWS have been developed and applied in many fields, including food, agriculture, and natural disasters (e.g., tsunamis). The purpose of EWS is to reduce vulnerability and increase preparedness (WHO 2004). Over recent years, model-driven EWS have been attempted increasingly in epidemiology and public health, particularly for the control and prevention of infectious diseases. In general, EWS are regarded as management tools to predict outbreaks of infectious diseases so that health authorities can prepare for the epidemics and act accordingly to mitigate against or avoid the impact of these diseases (Teklehaimanot et al. 2004a, 2004b). The development of spatiotemporal models has been rapidly evolving in the area of control and prevention of MBDs. Some models have been successfully developed to predict the likelihood of MBD epidemics using weather and environmental data (Teklehaimanot et al. 2004a, 2004b; Thomson et al. 2006).

RRV disease has strong spatial and temporal patterns, because mosquito density and longevity depend on a number of environmental and social factors (e.g., temperature, precipitation, mosquito-breeding habitats, vector control programs) (Hu et al. 2005; Kelly-Hope et al. 2004a; Tong et al. 2001). The pattern of RRV and other MBDs is likely to change with the changing socioecologic conditions (e.g., urbanization, increasing travel, global climate change) (Russell 1998; Tong et al. 2001; Woodruff et al. 2006). Most published reviews indicate that RRV may be influenced by climate change, and the impact may vary from region to region (Harley et al. 2001; Russell 2002). However, although RRV is the most common and widespread MBD in Australia and some Pacific island nations, little research has been conducted to develop an integrated EWS to forecast the likelihood of RRV outbreaks in different socioecologic regions, particularly in new urban estates and popular tourist areas.

Thus, there is a need to facilitate short-term epidemic forecasting and to improve scenario-based predictive modeling for the control and prevention of RRV and other MBDs to enhance biosecurity, to better adapt to rapid socioecologic changes, and to minimize the adverse public health impact of these changes. Climate variability strongly affects the replication of the virus, the breeding, abundance, and survival of the mosquito species, the breeding patterns of major hosts, and human behavior (Harley et al. 2001; Russell 1998; Tong 2004). These variables can be modeled to predict the onset and severity of disease epidemics at different temporal and spatial scales. Further, it is anticipated that the properly developed EWS for RRV disease transmission will improve our understanding of biologic/ecologic mechanisms of disease outbreaks and may have wide applications in planning RRV and other disease control and risk management programs.

Disease control measures such as EWS have great public health implications. Comprehensive and interdisciplinary EWS can greatly assist in improving vector control and personal protection to reduce the likelihood of RRV epidemics, as the previous models generally provided 1–3 months of advance warning (Hu et al. 2005, 2006a, 2007; Tong et al. 1998, 2001, 2002, 2004, 2005; Tong and Hu 2002). For example, differentially increasing insecticide spraying in high-risk areas during high-risk periods and decreasing it in low-risk areas and periods will improve cost effectiveness of vector control operations. If anticipating an outbreak of RRV disease, decision makers in disease control programs (e.g., communicable disease managers) can increase vigilance (e.g., by alerting district health offices), enhance vector control activity, request more frequent reporting to facilitate early identification of any problem, and initiate community education programs promptly in the affected areas. Additionally, the novel methods developed in these studies are also potentially useful for other MBDs (e.g., malaria, dengue fever, Japanese encephalitis) and may assist health authorities in determining public health priorities more wisely and using resources more efficiently, especially in the context of climate and sea level changes. Although EWS have significant implications in disease control and surveillance, such measures have not been formally implemented in Australia. There is an urgent need to bridge the gaps between science and policy.

## Conclusions

A growing body of evidence suggests that, even though many factors can affect the transmission cycles of RRV disease, climate and tidal variables (including rainfall, temperature, and tidal levels) and mosquito density are generally important environmental predictors of RRV disease. However, the nature and magnitude of such associations may vary with geographic area and socioenvironmental condition, and thus, further in-depth research on socio-environmental change and RRV transmission is required at local, regional, and national levels.

An assessment of factors predicting RRV disease transmission will help local authorities identify periods of high risk, optimizing the provision of additional mosquito control measures and community education. Climate data are relatively cheaper and easier to collect than other data (e.g., mosquito density and species). It is possible to improve the effectiveness of public health responses through the prediction of RRV epidemics with the appropriate integration and analysis of weather forecast, social and environmental factors, and disease surveillance data. However, the relationship between climate variables and RRV needs to be viewed within a wider context of other social and environmental changes. To advance this field, key research priorities include

- Methodologic development for quantifying the relative importance of climate variability/change and other social and environmental factors in outbreaks of RRV disease
- Further in-depth research on socioenvironmental change and RRV transmission at different levels, taking into account the nature and magnitude of the relationship between climate variability and RRV transmission that may vary with geographic area and socioenvironmental condition (it is important to integrate both climatic and non-climatic factors in the development of EWS)
- Development of scenario-based risk assessment models to project possible impacts of climate change on RRV transmission from local to national
- Develop different public health intervention strategies to control and prevent this disease at different levels to address RRV epidemiologic patterns throughout Australia (it is important to evaluate what public health intervention strategies are effective to control and prevent this disease at different levels).

The prospect of a changing regional climate is likely to affect the seasonal and geographic distribution of RRV. Projected anthropogenic global climatic change may result in an increase in RRV infections. The details of these changes will be difficult to predict at a regional level, but the development of EWS should allow actions to reduce the incidence of the disease, offsetting any increases arising from the changing climate. Thus, the development of these systems can help us cope with RRV in the current climate and help us adapt to the consequences of climate change in the future, at minimal cost. Of course, care will need to be taken to ensure that climate changes do not, of themselves, change the underlying climate/heath/societal relationships that form the basis of the EWS.

Clearly, a concerted, interdisciplinary, multisectoral effort is required to design and implement these research activities. We anticipate that climate/RRV research will provide an inspirational opportunity for researchers, policy makers, and other stakeholders to work together more closely to control and prevent this widespread disease.

## Figures and Tables

**Table 1:** Major population-based studies on climatic, social, and environmental factors and RRV disease.^a^

Study	Location (study period)	Research design	Key statistical method	Major finding	Comment
Tong et al. 1998	Toowoomba, Queensland (1986–1995)	Ecologic study	Spearman’s rank correlation	Increases in temperature (particularly in minimum temperature), rainfall, humidity, and SOI were positively associated with the incidence of RRV disease	Only one inland city was included
Tong and Hu 2001	Cairns, Queensland (1985–1996)	Time-series analysis	ARIMA model	Rainfall and relative humidity appeared to play significant roles in the transmission of RRV disease in Cairns	Only one coastal city was included
Tong et al. 2002	Queensland (1985–1996)	Time-series analysis	ARIMA model	Overall, rainfall, temperature, and tidal levels were important environmental determinants in the transmission cycles of RRV disease across Queensland	The magnitude of the climate–RRV relationship varied across the eight major cities in Queensland
Done et al. 2002	Queensland (1991–1997)	Time-series analysis	Correlograms and periodograms	The quasi-biennial cycle accounts for 77% of the variance in RRV cases.	Spatial variation was not examined
Tong and Hu 2002	Coastline and inland regions in Queensland (1985–1996)	Time-series analysis	Poisson regression	Maximum temperature exhibited a greater impact on the RRV transmission in coastline than in inland cities; minimum temperature and relative humidity seemed to affect the RRV transmission more at the inland than the coastline.	The relation between climate variables and RRV needs to be viewed within a wider context of other socio- environmental variability
Woodruff et al. 2002	Southeastern Australia (1991–1999)	Ecologic study	Logistic regression	Early warning of weather conditions conducive to outbreaks of RRV disease is possible	The sensitivity of the model varied with area
Tong et al. 2004	Townsville region (1985–1996)	Time-series analysis	SARIMA model	Rainfall, high tide, and maximum temperature were likely to be key determinants of RRV transmission in the Townsville region	Spatial variation was not examined
Hu et al. 2004	Brisbane (1985–2001)	Time-series analysis	SARIMA model	Monthly precipitation was significantly associated with RRV transmission but had no significant association for other climate variables (e.g., temperature, relative humidity, high tides)	This is a broad, ecologic assessment at the city level; more detailed risk assessment at community and individual levels may also be required
Kelly-Hope et al. 2004a	Four geoclimatic regions in Australia (various periods from 1971 to 1997)	Ecologic study	Descriptive statistics	Rainfall in outbreak years tended to be above average and higher than rainfall in nonoutbreak years. Overall temperatures were warmer during outbreak years; however, seasonal and monthly trends differed across geoclimatic regions of the country	It is unclear how RRV outbreak years were defined across four regions
Kelly-Hope et al. 2004b	Australia (1896–1998)	Analysis of historical reports	Descriptive statistics	The magnitude, regularity, seasonality, and locality of outbreaks ranged widely; environmental conditions act differently in tropical, arid, and temperate regions. Overall, rainfall seems to be the single most important risk factor.	Information bias is likely to occur on how to define and record outbreaks for such a long period
Tong et al. 2005	Brisbane (1998–2001)	Time-series analysis	Poisson regression	There were complex interrelationships between rainfall, mosquito density, and RRV transmission	Only one metropolitan city was included
Gatton et al. 2005	Queensland (1991–2001)	Ecologic study	Logistic regression	The variables identified as important in predicting RRV disease outbreaks differed between regions and also between summer and autumn	Selection bias might occur in choosing different stations for different local government areas
Hu et al. 2006a	Brisbane (1998–2001)	Time-series analysis	Polynomial distributed lag and SARIMA models	Both rainfall and mosquito density were strong predictors of the RRV transmission	Only one metropolitan city was included.
Woodruff et al. 2006	Western Australia (1991–1999)	Ecologic study	Logistic regression	Mosquito surveillance data could increase the accuracy of disease prediction models	Only a temperate region was included
Jacups et al. 2008	Darwin region (1991–2006)	Time-series analysis	Poisson regression	The best global model included rainfall, minimum temperature, and three mosquito species and can accurately predict RRV infections throughout the year in the Darwin region	Only a tropical region was included

Abbreviations: ARIMA, auto-regressive integrated moving average; SARIMA, seasonal auto-regressive integrated moving average; SOI, Southern Oscillation Index.

a By chronological order of publication.
